# Dysfunctional Activation of Neurotensin/IL-8 Pathway in Hepatocellular Carcinoma Is Associated with Increased Inflammatory Response in Microenvironment, More Epithelial Mesenchymal Transition in Cancer and Worse Prognosis in Patients

**DOI:** 10.1371/journal.pone.0056069

**Published:** 2013-02-13

**Authors:** Jinpu Yu, Xiubao Ren, Yongzi Chen, Pengpeng Liu, Xiyin Wei, Hui Li, Guoguang Ying, Kexin Chen, Hans Winkler, Xishan Hao

**Affiliations:** 1 TMUCIH-JnJ joint laboratory, Tianjin Medical University Cancer Institute and Hospital, Key Laboratory of Cancer Prevention and Therapy, Tianjin, China; 2 Department of Immunology, Tianjin Medical University Cancer Institute and Hospital, Key Laboratory of Cancer Prevention and Therapy, Tianjin, China; 3 Laboratory of Cancer Cell Biology, Tianjin Medical University Cancer Institute and Hospital, Key Laboratory of Cancer Prevention and Therapy, Tianjin, China; 4 Department of Epidemiology, Tianjin Medical University Cancer Institute & Hospital, Key Laboratory of Cancer Prevention and Therapy, Tianjin, China; The University of Hong Kong, China

## Abstract

**Aim:**

To investigate the role of neurotensin (NTS) in hepatocellular carcinoma (HCC) sub- grouping and the clinical and pathological significance of activation of NTS/IL-8 pathway in HCC.

**Methods:**

The genome-wide gene expression profiling were conducted in 10 pairs of cancer tissues and corresponding normal adjacent tissues samples using Affymetrix GeneChip® Human Genome U133 Plus 2.0 microarray to screen differentially expressing genes and enrich dysfunctional activated pathways among different HCC subgroups. The levels of NTS protein and multiple inflammation and epithelial mesenchymal transition (EMT) related proteins, including IL-8, VEGF, MMP9, CD68, E-Cadherin, β-Catenin and Vimentin were examined in 64 cases of paraffin-embedded HCC samples using immunohistochemistry (IHC) staining method. The clinical outcome and overall survival (OS) were compared.

**Results:**

A subgroup of HCC characterized by up-regulated NTS expression was accompanied by up-regulated inflammatory responses and EMT. The direct interaction between NTS and IL-8 was identified by pathway enrichment analysis. Significantly increased IL-8 protein was confirmed in 90.91% of NTS^+^ HCC samples and significantly positively correlated to the levels of NTS protein in cancer tissues (P = 0.036), which implied activation of NTS/IL-8 pathway in HCC. The levels of VEGF and MMP9 correlated with co-expression of NTS and IL-8. Increased infiltration of CD68^+^ macrophages and more cancer cells displaying EMT features were found in NTS^+^IL-8^+^ samples. The co-expression of NTS and IL-8 in cancer significantly correlated with the clinical outcomes, as the mortality rate of NTS^+^IL-8^+^ HCC patients is 2.5-fold higher than the others after the surgery (P = 0.022). Accordingly, the OS of NTS^+^IL-8^+^ HCC patients significantly decreased who are under a higher hazard of death at an expected hazard ratio (HR) of 3.457.

**Conclusion:**

Dysfunctional activation of the NTS/IL-8 pathway was detected in HCC which is associated with increased inflammatory response in microenvironment, enhanced EMT in cancer, and worse prognosis in HCC patients.

## Introduction

Hepatocellular carcinoma (HCC) is one of the most commonly diagnosed cancers with the mortality rate ranking at the third worldwide [1]. Due to the high frequency of local metastasis and recurrence after surgery, the 5-year survival rate in HCC patients is less than 5%. Therefore, identification of biomarkers significantly relate to invasion and metastasis of HCC cells and elucidation of the molecular mechanisms for developing novel therapeutic approaches are essential for better HCC treatment.

Inflammation represents a major pathologic factor for malignancies in the occurrence and progression of HCC. Persistent infections of hepatitis viruses and chronic cirrhosis induced by non-viral factors, such as alcohol and inherited metabolic disease in liver, have been shown to lead to long-lasting infiltration of inflammatory cells and formation of HCC [2]. According to the “immune-editing” theory [3], several types of inflammatory cells in cancers, including tumor associated macrophages (TAMs) [4], regulatory dendritic cells (DCs) [5], polymorphonuclear neutrophils (PMNs) [6] and mast cells [7], produce inflammatory cytokines and chemokines that promote the growth and metastasis of cancers [8]. In addition to infiltrating immunocytes, it has been reported that multiple cytokines produced by malignant cells contribute to the development and maintenance of a local inflammatory microenvironment [9], [10]. However, concrete mechanisms regulating inflammation-related development and progression of HCC are still unclear.

Considering the complexity of hepato-carcinogenesis, a large number of genes might be involved in inflammation-related development and progression of HCC. Therefore, high-dimensional array technologies and analytical high-throughput genome-wide gene expression profiling may provide a systematic and efficient approach to discover new candidate genes which could serve as potential therapeutic targets or predictive biomarkers for HCC patients [11]. Currently, a large number of microarray studies conducted in HCC have shown different outcomes, including generation of characteristic gene signatures for molecular classification of HCC and development of novel predictors for metastasis, recurrence and outcome of HCC [12], [13]. However, because of the low reproducibility and limited predictive accuracy of each reported system in different cohorts, the ongoing challenge is to identify and characterize clinically relevant genes that can serve as classification biomarkers and therapeutic targets simultaneously based on known pathway alterations in HCC.

Therefore, in this study we conducted comprehensive expression analysis of a total of 41 cases of genome-wide gene expression profiling data from HCC tissues (10 from in-house database, 31 from the public database GEO). We used a specific, unsupervised filtering method–Spectral Map Analysis (SMA) to distinguish subgroups with different genetic features among HCC samples and screen for prevalent candidate genes differentially expressed in different subgroups. In order to demonstrate the clinical significance of target genes, we performed immunohistochemistry (IHC) staining to determine their expression in 64 cases of primary HCC tissues and evaluated the relevance among the protein expression, clinical features, and the prognosis of these patients. We found a distinct HCC subgroup characterized by increased NTS expression which was accompanied by increased inflammatory responses and up-regulated epithelial mesenchymal transition (EMT) related signal pathways. Dysfunctional activation of the inflammation-related NTS/IL-8 pathway was identified by pathway enrichment analysis and IHC staining method, in which synchronous increase of NTS and IL-8 in cancer cells was detected in 90.91% NTS^+^ HCC samples. Co-expression of NTS and IL-8 positively correlated with increased expression of VEGF and MMP9 in cancer. Increased infiltration of CD68^+^ TAMs and higher number of cancer cells displaying EMT-related features were found in NTS^+^IL-8^+^ samples. Furthermore, co-expression of NTS and IL-8 in HCC significantly correlated with worse prognosis and shorter survival in patients after surgery. These results implied that dysfunctional activation of NTS/IL-8 pathway in HCC associates with increased inflammatory response in microenvironment, enhanced EMT in cancer and worse prognosis of patients. Thus, intervention of NTS/IL-8 pathway may be proposed as a novel strategy to reducing local inflammation and intervening cancer invasion which had the potential to serve as a promising predictive biomarker and/or therapeutic target for HCC.

## Materials and Methods

### Patient Information

A total of 64 cases of HCC patients who were treated with partial liver resection surgery at the Department of Hepatobiliary Oncology of Tianjin Cancer Institute & Hospital from November 2007 to July 2009 were enrolled in this study. These patients included 58 males and 6 females, with a mean age of 54.44 years (range: 35 to 71 years). The patients were pathologically diagnosed with HCC at histological grade I (n = 4), grade II (n = 36) and grade III (n = 24), and classified as stage I (n = 8), stage II (n = 44), and stage III (n = 12). No prior treatments (including chemotherapy or radiotherapy) were conducted before the liver resection surgery. Fresh tissue samples from 10 patients, including primary tumors and normal adjacent tissues, were collected and immediately stored at −80°C for microarray analysis. These 10 patients included 8 males and 2 females, with a mean age of 54.30 years (range: 38 to 71 years) and were pathologically diagnosed with HCC of histological grade II (n = 6) and grade III (n = 4), and classified as stage I (n = 3), stage II (n = 6), and stage III (n = 1). The paraffin embedded tissues from 64 patients were collected for pathological analysis. This project was approved by the Ethics Committee of Tianjin Medical University. All experiments were performed in accordance with the principles of Declaration of Helsinki. Written consents were obtained from each patient.

### RNA Isolation

Total RNA was extracted from the cancer tissues and adjacent normal tissues using the RNeasy® Mini Kit (Qiagen, Valencia, CA) following the manufacturer’s protocol. Integrity of the RNA was determined by electrophoresis. The concentration and purity of RNA were analyzed by spectrophotometry (Nanodrop8000, Thermo Scientific, MA, USA). RNA samples were stored at −80°C.

### Microarray Data Generated From the in-house Samples

RNA (250 ng) was reverse transcribed into cRNA using GeneChip® 3′ IVT Express Kit (Affymetrix, Santa Clara, CA, USA) and purified using BioMek® Fx workstation (Beckman Coulter, CA, USA). Afterwards, the purified cRNAs were fragmented into 35 to 200-nt long fragments and hybridized to the GeneChip® Human Genome U133 Plus 2.0 microarray chips (Affymetrix, Santa Clara, CA, USA) using standard protocols. The chips were washed in the GeneChip® Fluidics Station (Affymetrix, Santa Clara, CA, USA) and subsequently scanned using the GeneChip® Scanner 3000(Affymetrix, Santa Clara, CA, USA). Raw data were obtained using the GCOS software and analyzed using R2.9.0 software and Bioconductor 2.4.

### Microarray Data Downloaded From the Gene Expression Omnibus (GEO) Database

Thirty-one cases of gene expression profiling data of Asian HCC patients on the identical Affymetrix HGU133 plus 2.0 arrays were obtained from the Gene Expression Omnibus (GEO) database: GSE2109, GSE6465 [14], and GSE6222 [15].

### Quality Control of the Microarray Data

**Figure 1:**
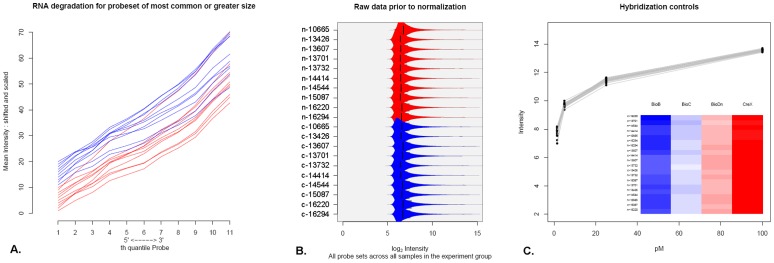
Quality control test of microarray was conducted for 20 cases of HCC samples and according normal adjacent tissues using the SimpleAffy package of Bioconductor 2.4. A-C). The QC results demonstrated that all RNA samples are in good quality without degradation, the signal intensities of each samples on chips are comparably distributed, and the hybridization signals of specific control genes are overlapped among chips. (A) Degree of RNA degradation. (B) Signal intensity of primary data. (C) Signal intensity of control genes.

After the raw data were achieved, a quality control (QC) of microarray data was conducted to evaluate the eligibility of the raw data. The QC test of 20 cases of HCC samples and according normal adjacent tissues was conducted using the SimpleAffy package of Bioconductor 2.4 in which normal RNA degradation, comparable distribution of signal intensities, and overlapped hybridization signals of specific control genes were confirmed (Figure 1A–C).

### Bioinformatics Analysis of the Microarray Data

Raw data of 10 pairs of in-house samples was preprocessed using GC-RMA method to conduct background correction and normalization. Summarization was done with median polish, using the hgu133plus2entrezg.13.0.0 CDF [16] and the preprocessed expression dataset (eSet) were filtered on I/NI calls [17]. All informative genes of 20 cases of experimental hepatic samples (10 cancer tissue samples and 10 adjacent normal tissue samples) were collected to generate corresponding spectral maps to distinguish the most significant diversity of gene expression profiles among samples. Spectral Map Analysis (SMA) is a multivariate projection method that allows reduction of the complexity of high dimensional data, in which a bi-plot is created by the first two principal components displays the maximal separation of both the transcripts and the samples which provides means to visually inspect and identify clusters of transcripts and/or subjects in the data [18]. In order to increase the credibility of results, other 31 cases of raw data of primary hepatic cancer tissue samples based on the same Affymetrix chip platform were downloaded from GEO and integrated with primary in-house raw data to conduct SMA. Subgroups consisted of different cluster of HCC samples were separated in SMA and differentially expressed genes in each subgroup were identified using the Linear Models for Microarray Data algorithm (LIMMA) [19]. For the spectral map and LIMMA analysis, the Bioconductor A4 package [20] was used. The criteria for differential expression were defined as a difference greater than 3 fold and *P*<0.05. The top 150 differentially expressed genes of the different HCC subgroups were separately entered into MetaCore™ 2.5 (GeneGo, Carlsbad, CA), Pathway Studio^®^ 6.0 (Ariadne Genomics, Rockville, MD, USA) and IPA^®^ 1.0 (Ingenuity Systems, Redwood City, CA, USA) software to screen for the most correlative signaling pathways involved in subgrouping and to construct a visual regulatory network [21].

### Pathological Validation of Differentially Expressed Genes

The expression and cell location of differentially expressed genes *in situ* were examined using IHC staining method among 64 cases of HCC cancer tissues and corresponding normal adjacent tissues. The inflammatory response in microenvironment was evaluated by comparing the expression of multiple inflammation- related proteins (including chemotaxin IL-8, angiogenic growth factors VEGF and matrix metalloproteinase MMP9) and the number of infiltrated CD68^+^ TAMs. The EMT phenotypic alteration of HCC cells was determined by loss of E-Cadherin on membrane and accumulation of β-Catenin and Vimentin in cytoplasm.

For IHC, samples were heated for 0.5 h at 56°C, de-paraffinized in xylene and rehydrated through graded alcohol. Antigens were retrieved by heating in citrate buffer (pH 6.0) for 20 minutes. Endogenous peroxidase activity was quenched in a bath of methanol and hydrogen peroxide for 30 minutes. All samples were incubated overnight at 4°C with mouse anti-human NTS, IL-8, VEGF, MMP9, CD68, E-Cadherin, β-Catenin and Vimentin monoclonal antibodies (Santa Cruz, CA, USA) at concentrations of 1∶1000, and a biotinylated secondary antibody (goat anti-mouse IgG, Santa Cruz, CA, USA) labeled with streptavidin-horseradish peroxidase (HRP) using a DAB staining kit (Maixin Biotechnology, Fuzhou, China). For negative control, the primary antibody was substituted with mouse isotype IgG1. Positive cells were stained brownish yellow in the cytoplasm or on the cell membrane. An Olympus BX51 microscope was used for image acquisition.

Five representative high-power fields (400× magnification) for each tissue section were selected for histology evaluation. For each protein, two parameters: Positive Rate (PR) and Staining Intensity (SI) were used to describe the expression based on the extensity and intensity of positively stained cells in the samples. PR is the percentage of positively stained cells in cancer tissues, in which the percentage ≤ 10% was defined as negative(scored as 0), 11–30% as positive of low frequency(scored as 1), 31–60% as positive of medium frequency(scored as 2), and >60% as positive of high frequency(scored as 3). SI is the ranked staining intensity of positively stained cells in HCC samples, which ranged from 0 to 3 and represented negative, dim positive, mediate positive and strong positive. Since the expression of certain protein was comprehensively evaluated based on both extensity and intensity, the sum of both parameters gave the final scores of each protein marker in each HCC sample, in which the final score <4 was defined as low/negative expression, and the final score ≥4 was defined as high expression.

### Statistical Analysis

All quantitative data were presented as mean ± standard deviation (SD). The statistical analysis was performed using the SPSS 16.0 software package. The chi-square (χ^2^) test was used to compare PRs and SIs among 64 cases of cancer tissues which were divided into different groups according to gender, age, status of HBV infection, previous history of smoke and alcohol, disease stage and clinical outcome after surgery. The Spearman’s rank-order test and linear regression analysis was used to assess the correlations between the protein levels of NTS and IL-8. The unpaired T test was used to compare the difference of VEGF, MMP9, CD68, E-Cadherin, β-Catenin and Vimentin expression between HCC samples expressing different levels of NTS and IL-8. The cumulative survival was determined by the Kaplan-Meier method, and the univariate survival analysis between different protein marker and overall survival (OS) of HCC patients was performed using the log-rank test. The multivariate survival analysis was performed using the Cox proportional hazard model and a forward stepwise procedure to screen independent prognostic factors. The level of statistical significance was set at *P*<0.05.

## Results

### A Subgroup of NTS High-expressing HCC is Identified Using Unsupervised Filtering Analysis of 20 Cases of Microarray Data from in-house Fresh Tissues

**Figure 2:**
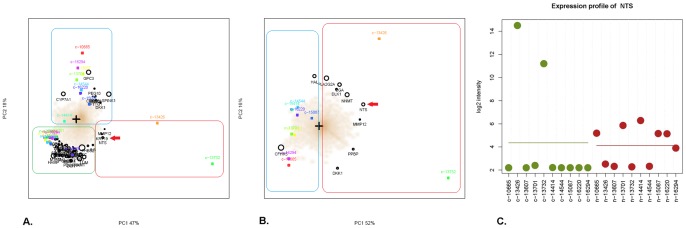
A subgroup of NTS high expressing HCC is identified from the gene expression profiling data of 10 pairs of in-house HCC tissues and corresponding adjacent normal tissues. Different subgroups of HCC samples were separated by the unsupervised filtering analysis SMA algorithm. In SMA, the sample names were shown in colorful squares. The genes were depicted in black circles and the size of the circle reflected the mean expression level of the gene over all samples. The subgroups of samples were separated using rectangles in different color, and the characteristic gene signature of certain subgroup was included in the corresponding rectangle as well. A). In Figure 2A, 3 subgroups of 20 cases of samples with distinct expression profiling features are separated along the X(PC1) axis and Y(PC2) axis. PC1 contributes 47% of variability and PC2 contributed 18% of variability in the gene expression profiling data of all 20 samples. The most significantly differentially expressed genes in each subgroup were listed in the same direction and included in the corresponding rectangle as well. Two HCC samples (c-13426 and c-13732, red rectangle) were distinguished from the others 8 cases of HCC samples (blue rectangle) and 10 cases of corresponding adjacent normal tissues (green rectangle) in the spectral map. NTS (red arrow), KRT19 and MMP12 were screened out as highly expressed genes in these 2 samples. B). The SMA was repeated in 10 cases of HCC samples which were divided into 2 subgroups scattering around the central marker “*+*”. Two HCC samples (c-13426 and c-13732, red rectangle) were separated out of the others 8 cases of HCC samples (blue rectangle), in which the NTS gene showed relatively higher expression in these 2 samples (red arrow). C). Quantity of NTS expression at mRNA level in the HCC tissues and adjacent normal tissues. The green plots represent cancer tissue samples, and the red plots represent normal adjacent tissue samples.

Using SMA, different subgroups of samples were separated according to the diversity of gene expression profiles of all 20 samples. In the biplot, all samples were divided into different subgroups scattering around the central marker “*+*”, and the most significantly differentially expressed genes in the certain subgroup of samples were listed in front of the certain subgroup which are considered as potential signatures for the separation of each subgroup from the other samples. The sample names were shown in colorful squares. The genes were depicted in black circles and the size of the circle reflected the mean expression level of the gene over all samples. The subgroups of samples were separated using rectangles in different color, and the characteristic gene signature of the certain subgroup was included in the corresponding rectangle as well (Figure 2A–B). Here, microarray data from 20 cases of experimental samples (10 cancer tissues and 10 normal tissues) were enrolled to conduct SMA. In Figure 2A, 3 subgroups with distinct expression profiling features are separated along the X(PC1) axis and Y(PC2) axis. PC1 contributes 47% of variability and PC2 contributed 18% of variability in the gene expression profiling data of all 20 samples. All cancer samples were separated from the normal adjacent tissue samples (green rectangle) on the different side of the spectral map, and two HCC samples (c-13426 and c-13732, red rectangle) were distinguished from the other cancer samples(blue rectangle), which located at the low part of the right side in the spectral map. In the top 50 differentially expressed genes between cancer and normal tissues, three genes: neurotensin (NTS, red arrow), keratin 19 (KRT19) and matrix metallopeptidase 12 (MMP12) were screened out for their high expression in 2 cancer samples (c-13426 and c-13732) (Figure 2A).

**Figure 3:**
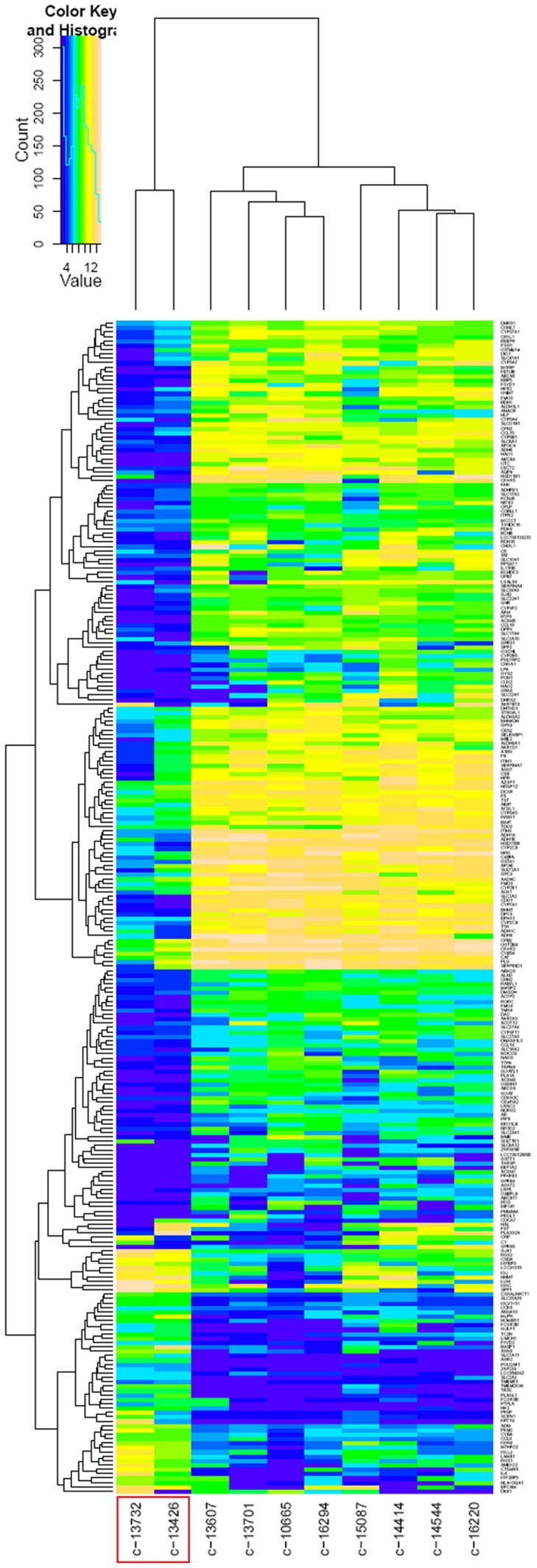
Genes differentially expressed between NTS high and low expressing HCC tissues were screened out. A total of 1274 genes differentially expressed between the NTS high and low expressing HCC samples were identified using LIMMA analysis, in which 579 genes were up-regulated and 695 genes were down-regulated. Hyper-geometric pathway enrichment analysis showed that the down-regulated genes are mainly involved in coagulation and metabolism, whereas the up-regulated genes contribute to inflammation-related biological progresses and HIF-1α and/or Wnt/β-catenin mediated EMT processes.

In order to identify differences of the gene expression profiling features between these 2 cancer samples and the other 8 cancer samples, microarray data from 10 cases of HCC samples (only cancer tissue) were used to conduct SMA separately. All samples were distinctly separated into 2 subgroups: 8 samples with similar expression features that are concentrated on the left side(blue rectangle), as opposed to two samples (c-13426 and c-13732, red rectangle) that cluster on the right. Among the top 10 differentially expressed genes between 2 subgroups, the NTS gene showed relatively higher expression in c-13426 and c-13732 (red arrow, Figure 2B).

Therefore, the expression of NTS in these hepatic cancer samples(green plots) as well as the corresponding adjacent normal tissues(red plots) was examined at the RNA level (Figure 2C). The result reveals that NTS is over-expressed in the two liver cancer samples (c-13426 and c-13732) compared to the other 8 HCC samples and all adjacent normal tissues samples. We therefore grouped the carcinoma samples according to their NTS expression: the high-NTS-expression group (c-13426 and c-13732) and low-NTS-expression group (c-10665, c-13607, c-13701, c-14414, c-14544, c-15067, c-16220, c-16294).

### Up-regulated NTS Gene Expression is Correlated to Activation of Inflammation and EMT -related Pathways after Pathway Enrichment

LIMMA analysis was used to identify a total of 1274 genes that were significantly differentially expressed between the NTS high and low expressing samples (*P*<0.05, Log FC >1.5; Fig. 3). When compared to the NTS low-expressing group, 579 genes were up-regulated and 695 genes were down-regulated in the 2 NTS high-expressing samples (red box in Fig. 3). Hyper-geometric pathway enrichment analysis conducted in Pathway Studio^®^ showed that the genes with down-regulated expression are mainly involved in coagulation and lipid metabolism, whereas those with up-regulated expression contribute to inflammation-related biological progresses, including the remodeling of the extracellular matrix (ECM), cell adhesion, leukocyte migration, and angiogenesis (Table 1). Additional pathway enrichment analysis in MetaCore™ and IPA^®^ confirmed the finding that the high NTS expression was closely related to inflammation-related pathways, including hepatic fibrosis/hepatic stellate cell activation, ECM remodeling, chemokines and adhesion, regulation of actin-based motility in the HCC tissue, and activation of HIF-1α and the Wnt/β-catenin-mediated EMT in HCC cells (data not shown).

**Table 1:** Pathways enriched from differentially expressed genes between HCC tissues of high and low NTS expression by PathwayStudio™.

Pathways enriched by Pathway Studio®	Number of overlapping genes	Overlapping genes	P value
***Down-regulated genes***			
Lipid metabolic process	48	CYP7A1,ACOX2,EHHADH,INSIG1,LRP5,HSD17B6,PLTP,HPGD,ACOT12,CYP3A4,PON1,TTPA,ACSL5,HSD11B1,ACAA2,ACSL6,LPA,SLC27A5,PLA1A,HADH,APOC4,BAAT,RDH16,APOE,ACSM5,ALDH3A2,ACSM3,HSD17B10,SLC27A3,SULT2A1,ABCB4,THRSP,SRD5A2,DGAT2,PLCL2,ACSL1,AKR1D1,ECH1,PMVK,ACADSB,AADAC,ACADL,FADS1,APOB,ACSM1,ECHDC2,CYP7B1,MBTPS2	3.184e−22
Fatty acid metabolic process	27	ACSM5,FABP4,ACOX2,PCCA,EHHADH,GPAM,ACSM3,SC4MOL,CYP4A11,HPGD,ACOT12,SLC27A3,CYB5A,CRYL1,ACSL5,HAO2,ACSL1,ACAA2,ACSL6,ECH1,SLC27A5,ACADSB,ACADL,HADH,BAAT,ACSM1,ECHDC2	1.623e−20
Steroid metabolic process	14	CYP7A1,INSIG1,HSD17B6,AKR1B10,SULT2A1,OSBPL6,HSD11B1,CYP3A5,AKR1D1,NR1I2,OSBPL3,APOB,CYP7B1,MBTPS2	1.932e−8
Aldehyde metabolic process	6	ALDH3A2,AKR7A3,AKR1B10,ALDH7A1,ADH4,ALDH1A1	1.547e−7
Blood coagulation	13	SERPINC1,F9,F5,F7,LMAN1,SERPIND1,F11,PLG,LPA,COCH,SERPINE1,F13B,F10	2.841e−7
Amino acid metabolic process	10	SLC7A5,SDS,CLN3,GLUD1,TAT,HMGCL,MAT1A,SLC7A2,OTC,ASNS	1.296e−6
Cholesterol metabolic process	10	CYP7A1,INSIG1,CYP27A1,PON1,TNFSF4,NR0B2,APOB,CYP7B1,APOE,MBTPS2	1.535e−5
Biotin metabolic process	3	PCCA,MCCC1,BTD	1.954e−5
Bile acid metabolic process	5	ACOX2,AMACR,SLC27A5,AKR1C1,BAAT	4.794e−5
Acyl-CoA metabolic process	6	EHHADH,ACOT12,SLC27A3,GLYAT,HMGCL,ACSL6	5.458e−5
***Up-regulated genes***			
Inflammatory response	21	CXCL2,CCL16,CCL5,KNG1,CCR5,TNFSF4,NFKBIZ,ATRN,IL8,BLNK,CCL25,CCL8,SPP1,CDO1,EPHX2,EVI1,AOX1,ALOX5AP,CYP4F11,CRP,CYP4F2	1.848e−4
Response to reactive oxygen species	4	CAT,ALDH3A2,SERPINE1,APOE	7.370 e−4
Acute-phase response	6	CEBPA,LBP,ASS1,HPR,CRP,BAAT	7.567e−4
Complement activation	7	MASP2,C4BPA,C8B,CRP,MBL2,C7,C6	9.159e−4
Chemotaxis	11	CXCL2,CCL15,CCL16,CCL5,SERPIND1,CCR5,C5AR1,IL8,CCL25,LECT2,CCL8	0.002178
Immune response	34	CXCL2,MASP2,COLEC12,CCL14,CCL16,CCL5,C4BPA,CCR5,TNFSF4,AQP9,FCGR2B,IL6R,C5AR1,HLA-DQA1,TCF19,MBL2,AZGP1,IL8,CCL25,CCL8,IER3,CCL15,DPP4,IGKC,HAMP,NEDD4,FCGR3B,RGS1,IGHM,C8B,GBP5,AFP,IGJ,CD1D	0.008114
Leukocyte migration	11	ICAM1,IL6,MMP9,PECAM1,VEGFA,ITGA2,THBS1,ADAM10,SELP,ITGB2,SELE	0.01
Angiogenesis regulation	9	Rhob,VASH2,AGGF1,AMOT,ANGPTL3, BTG1,ITGB2, IL1B,HIF1A	0.01
Cell adhesion	13	ADAM9,AGT,CD44,CDK5,COL3A1,COL5A3,CTGF,ECM2,CTNNB1,FN1,ITGA3,ITGA6, RHOA	0.01
Collagen fibril organization	8	COL11A1,COL1A1,COL2A1,COL3A1, COL5A1,SERP1NH1,TGFB2,TGFBR1	0.01

### A Subgroup of HCC High Expressing NTS was Confirmed in the Expanded Pool of 40 Microarray Data and Accompanied with Significantly Up-regulated Inflammation-related Pathways

**Figure 4:**
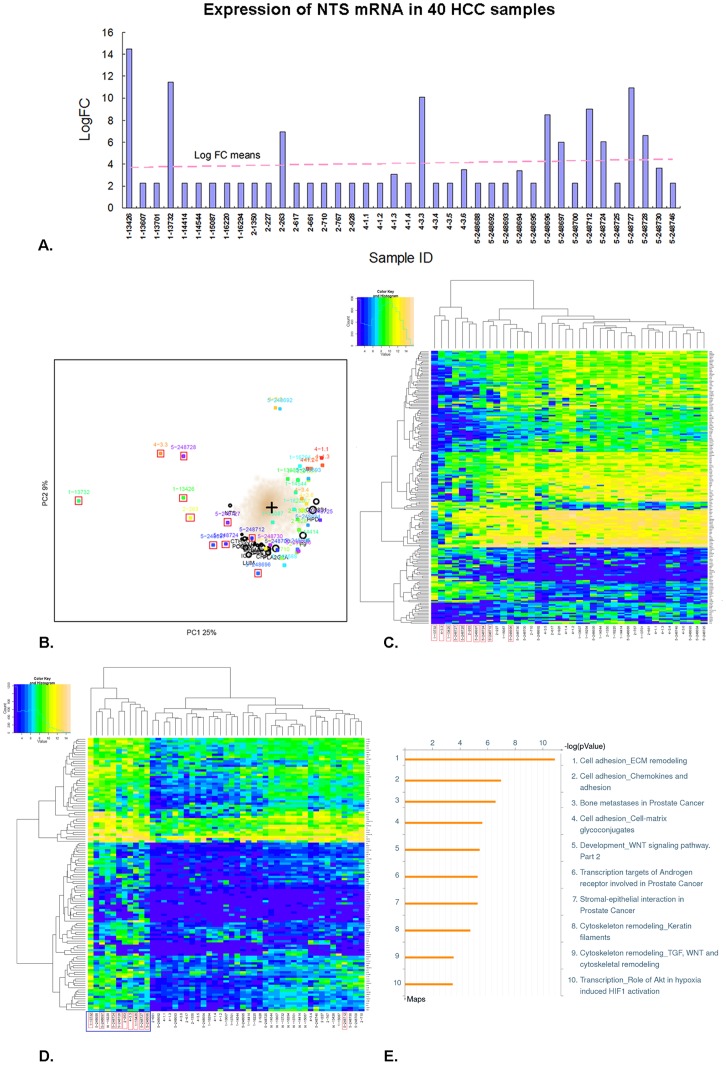
A subgroup of HCC high expressing NTS was confirmed in the expanded pool of 40 microarray data accompanied with significantly up-regulated inflammation-related pathways. NTS expression in 40 cases of integrated microarray data composing of 9 cases of in-house data and 31 cases of public GEO data. A subgroup of 10 cases of NTS high expressing HCC samples was separated out in the spectral map using the SMA algorithm. A). Quantity of NTS expression at the mRNA level in 40 HCC tissues. NTS was found to be elevated in 10 samples (1–13426, 1–13732, 2–263, 4–3.3, 5–248696, 5–248697, 5–248712, 5–248724, 5–248727, 5–248728). B). These 10 NTS high expressing samples(red box) clustered on the left side of the spectral map, in which NTS was shown as one of the top 20 highly expressed genes. C). LIMMA analysis was performed to identify the differentially expressed genes between NTS high and low expressing samples, and a panel of 457 gene was generated, including 166 up-regulated genes and 291 down-regulated genes. D). The LIMMA analysis was repeated after 10 normal adjacent tissues were integrated to screen NTS related differentially expressed genes. A list of 123 common up-regulated genes in NTS high expressing samples was generated which can distinctively distinguish NTS over-expressing cancer samples (red box) from the others in the heat map (blue box). E).Top 10 pathways dysregulated in NTS high expressing samples were enriched from the 123 common up-regulated genes. The inflammation-related processes and invasion-related signal pathways were up-regulated in NTS over-expressing tissues.

To further confirm our findings, we supplemented our in-house gene expression raw data with public profiling data of HCC tissues in Asian patients from the Gene Expression Omnibus (GEO) (GSE2109, GSE6222 and GSE9843), yielding 31 evaluable cancer samples. We integrated these 31 cases of raw data with the 10 primary cases of in-house data to conduct data processing and finally generated a qualified eSet of 40 cases (1 in-house sample was deleted for high batch effect). NTS was found to be elevated in 10 samples (1–13426, 1–13732, 2–263, 4–3.3, 5–248696, 5–248697, 5–248712, 5–248724, 5–248727, 5–248728, red box in Fig. 4B–D) as compared to the other 30 samples (Fig. 4A). SMA was conducted to distinguish clusters of transcripts and/or subjects in 40 microarray data. Similar to previous observation, the 10 samples with high NTS expression clustered together on the left side and the remaining 30 samples with low NTS expression on the right side of the unfiltered spectral map, and NTS was shown as one of the top 20 differentially expressed genes between these 2 clusters (Fig. 4B). This result indicates that NTS is a pivotal biomarker to distinguish different subsets of HCC samples of variable transcriptional features. Therefore, LIMMA analysis was performed to identify the differentially expressed genes between 2 clusters of samples with different expression level of NTS in which 457 genes were screened out, including 166 up-regulated genes and 291 down-regulated genes (*P*<0.05, log fold change (Log FC) >1.5, Fig. 4C).

Afterwards, 10 cases of normal adjacent tissues were integrated with 40 cases of tumor tissues to repeat the LIMMA analysis using NTS as the exclusive subgrouping factor. A list of 123 commonly up-regulated genes in high-NTS samples was generated from all 49 cases of samples which can distinctively distinguish NTS over-expressing cancer samples from the low expressing samples in the heat map (blue box in Fig. 4D). Therefore, the 123 genes were inputted into the MetaCore™ and IPA^®^ application software to enrich possible pathways affected by NTS and demonstrated that inflammation-related pathways, including ECM remodeling, chemotaxis and adhesion, cell-matrix glycol-conjugates, stromal-epithelial interaction and cytoskeleton remodeling were up-regulated in NTS-overexpressing tissues (Fig. 4E). Furthermore, 2 invasion-related pathways: HIF-1α signaling and Wnt/β-catenin signaling were up-regulated in NTS overexpressing tissues (Fig. 4E). Pathways enrichment analysis confirmed the finding that high NTS expression was closely related to the development of inflammatory response in microenvironment and partly associated with increased migration and invasion of tumor cells.

### Synchronous Increase of NTS and IL-8 Proteins in Cancer Cells Implied Dysfunctional Activation of the NTS/IL-8 Pathway in HCC

**Figure 5:**
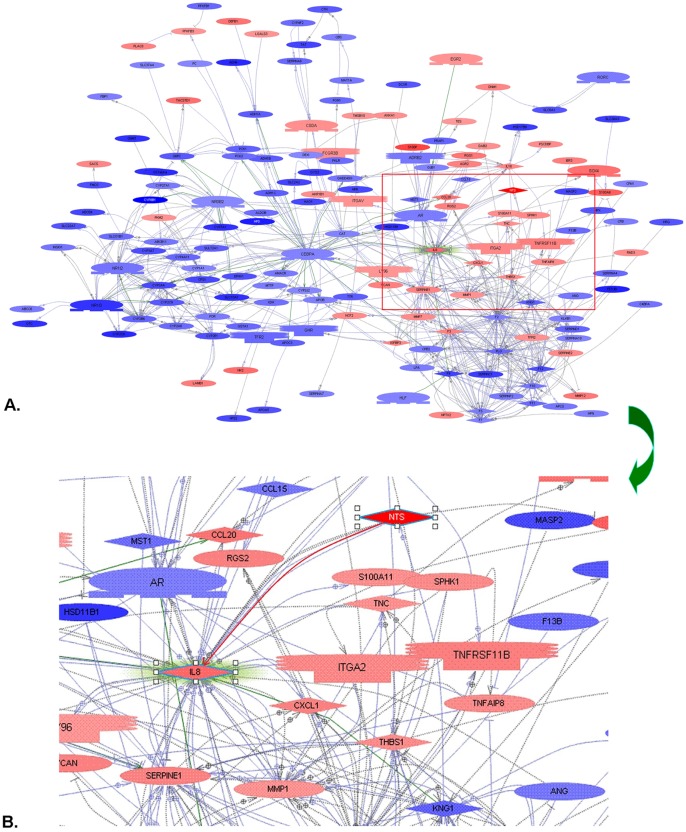
All significantly differentially expressed genes between high and low NTS samples were put into Pathway Studio^®^ to screen the candidates that were regulated by or interacted with NTS. The NTS/IL-8 pathway, a predominant inflammation-related pathway in colorectal carcinogenesis was distinguished. A). Up-regulated NTS expression closely correlated with increase of multiple inflammatory molecules. B). Direct interaction between NTS and chemotaxin IL-8 was confirmed.

For further analysis, we imported all significantly differentially expressed genes between NTS high and low expressing samples into Pathway Studio^®^ to screen the candidate genes that were regulated by or interacted with the NTS gene [22]. The direct interaction between NTS and IL-8 protein, defined as the NTS/IL-8 pathway, has been reported to induce chronic inflammation in colorectal disease and play a pivotal role in the development and progression of multiple cancer types except HCC [23]–[26] (Fig. 5A–B). Since IL-8 is a potent pro-inflammatory chemotaxis cytokine that directly regulates cell migration, angiogenesis and tumor invasion, we hypothesized that NTS promotes inflammation via an IL-8 dependent pathway.

**Figure 6:**
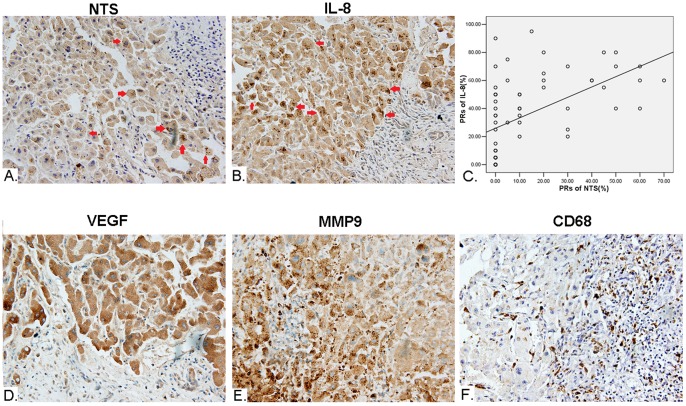
Synchronous increase of NTS and IL-8 proteins in cancer cells closely correlated with the up-regulated inflammatory response in microenvironment of HCC. The expression of multiple protein markers in HCC was detected in 64 cases of primary HCC tissues and corresponding normal adjacent tissues using IHC staining method. A). Ectopic expression of NTS (red arrowhead) is observed in 17.19% (11/64) of HCC tissues. B). Synchronous and consistent expression of IL-8 (red arrowhead) was observed in 90.91%(10/11) of NTS^+^ samples, which is much higher than the rate of 54.69% (35/64) among all HCC samples. C). The PRs of IL-8 significantly positively correlated to the PRs of NTS in cancer cells with a linear regression equations of Y = 28.213+0.456X (Y: PRs of IL-8; X: PRs of NTS) (R = 0.316, P = 0.011). D). The expression of VEGF is significantly higher in NTS^+^IL-8^+^ HCC samples compared to the others (P = 0.020). E). The expression of MMP9 is significantly higher in NTS^+^ and NTS^+^IL-8^+^ HCC samples(P = 0.004 and P = 0.0051). F). Increased infiltration of CD68^+^ TAMs was observed in NTS^+^IL-8^+^ HCC samples.

Therefore, the expression of NTS and IL-8 protein in HCC was detected in 64 cases of primary HCC tissues and corresponding normal adjacent tissues using IHC staining method. We found that the frequency of NTS expressing tissues among all HCC samples was 17.19% (11/64). The NTS positive staining cells are exclusively HCC cells, in which brown-yellow particles were observed in cytoplasm(red arrowhead, Fig. 6A). No positive staining was observed in normal adjacent tissues. The average PR among 11 positive samples is (50.00±11.62)% and the average SI is 2.91±0.3, which implied the expression of NTS in positive cancer tissues is relatively extensive and strong though the frequency of positive cancer tissues is relatively low. Consistently, brown-yellow particles were observed in cytoplasm of IL-8 positively stained HCC cells(red arrowhead, Fig. 6B). The frequency of IL-8 expressing tissues among all HCC samples was 54.69% (35/64), but the frequency of IL-8 expressing tissues in NTS positive samples is much higher, 90.91%(10/11), which implied that most of NTS positive HCC cells co-expressed IL-8.

We compared the expression and distribution of IL-8 protein between NTS high expressing (NTS^+^) samples and NTS low-expressing (NTS^−^) samples. The average PR of IL-8 among 11 cases of NTS^+^ samples is (50.91±13.57)%, significantly higher than that of (31.42±29.39)% among 53 NTS^−^ samples (P = 0.036), which implied that the expression of IL-8 was up-regulated in NTS high expressing HCC tissues. Furthermore, a scatter plot was generated to display the correlation between NTS and IL-8 protein levels in 64 samples using the curve estimation method in Regression Analysis. It’s demonstrated that the PRs of IL-8 significantly positively correlated to the PRs of NTS in cancer cells with a linear regression equations of Y = 28.213+0.456X (Y: PRs of IL-8; X: PRs of NTS) (R = 0.316, P = 0.011, Fig. 6C). Therefore, the synchronous increase of NTS and IL-8 proteins in 15.63%(10/64) HCC samples implied dysfunctional activation of the NTS/IL-8 pathway in a part of HCC.

### Co-expression of NTS and IL-8 is Associated with Increased Inflammatory Response in Microenvironment and Enhanced EMT in Cancer Cells

In order to determine if co-expression of NTS and IL-8 (NTS^+^IL-8^+^) in HCC tissues correlates with the development of a local inflammatory response in microenvironment, the expression of VEGF and MMP9 proteins, as well as infiltration of CD68^+^ TAMs were studied among 64 cases of primary HCC tissues and corresponding normal adjacent tissues. The positive rate of VEGF in cancer was 48.44% (31/64, Fig. 6D) and the positive rate of MMP-9 was 45.31% (29/64, Fig. 6E). We then compared the expression of both protein markers between NTS^+^ samples and NTS^−^ samples. The average PR of MMP9 in NTS^+^ samples is (49.09±25.57)%, significantly higher than that of (23.58±26.13)% among NTS^−^ ones (P = 0.004). In contrast, the difference of the PRs of VEGF between NTS^+^ and NTS^−^ samples is not significant, which is (46.36±32.95)% vs. (32.55±29.19)% (P = 0.167). However, the expression of VEGF is significantly higher in NTS^+^IL-8^+^ HCC samples compared to the others, which is (55.00±30.27)% vs. (31.02±28.92)% (P = 0.020). Simultaneously, increased infiltration of CD68^+^TAMs was observed in NTS^+^IL-8^+^ HCC samples (Fig. 6F). These results implied that dysfunctional activation of the NTS/IL-8 pathway was prone to induce secretion of multiple inflammatory cytokines and recruit CD68^+^ TAMs that promote development of inflammatory microenvironment in HCC.

**Figure 7:**
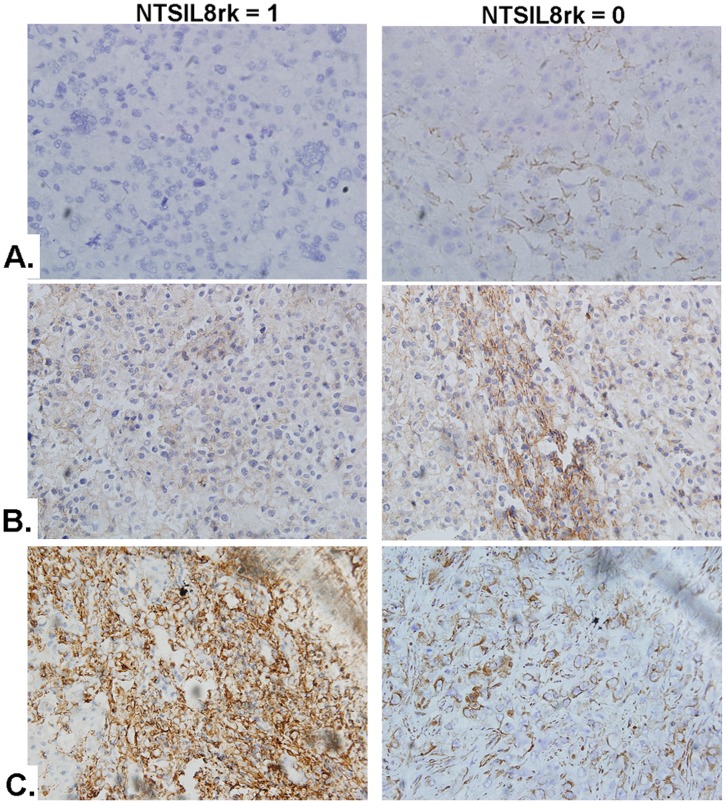
Co-expression of NTS and IL-8 associated with enhanced EMT in cancer cells. The expression of three EMT markers: E-Cadherin, β-Catenin and Vimentin was examined to evaluate the status of EMT in NTS^+^IL-8^+^ HCC tissues. A-C). Loss of expression of E-Cadherin on membrane and increased accumulation of β-catenin and Vimentin in cytoplasm was found in NTS^+^IL-8^+^ HCC samples. Statistical analysis showed that the expression of E-Cadherin and β-catenin significantly correlated with the level of IL-8 in HCC samples. A). E-Cadherin. B). β-catenin. C). Vimentin. Note: 0: Negative of co-expression of NTS and IL-8 in cancer tissues; 1: Positive of co-expression of NTS and IL-8 in cancer tissues.

Furthermore, three EMT markers, including E-Cadherin, β-Catenin and Vimentin were studied to evaluate the EMT status in NTS/IL-8 co-expressing HCC tissues. The characteristic features of EMT was found in NTS^+^IL-8^+^ HCC samples, including loss of membrane expression of E-Cadherin (Fig. 7A) and increased cytoplasmic accumulation of β-catenin and Vimentin (Fig. 7B–C). Statistical analysis showed that the expression of E-Cadherin and β-catenin significantly correlated with the level of IL-8 in HCC samples (Rcad = 0.299, Pcad = 0.002 for E-Cadherin; Rcat = 0.247, Pcat = 0.020 for β-catenin) which implied that up-regulated expression of IL-8 after dysfunctional activation of the NTS/IL-8 pathway might promote EMT in HCC cells.

### Synchronous Increase of NTS and IL-8 in HCC Correlates with Worse Aprognosis and Shorter Survival of Patients after Surgery

We compared the expression of NTS among patients with different gender, age, status of HBV infection, history of alcohol and smoke, disease stage, and clinical outcome. The levels of NTS expression didn’t vary significantly between male and female, elder and younger, HBV infected and uninfected patients. Besides that, no significance was identified between patients with and without history of alcohol consumption or smoke. But it’s evident that the expression levels of NTS correlated with the clinical outcomes although they didn’t vary significantly among HCC samples at different clinical stage. It was observed that near one third of HCC patients who passed away had NTS-overexpressing tumors, which is significantly higher than the percentage in alive HCC patients (31.58% vs. 11.11%, P = 0.047, Table 2). We further compared the clinical outcome of the patients co-expressing NTS and IL-8 with the others, and found 60%(6/10) of HCC patients bearing NTS^+^IL-8^+^ tumors died after the surgery, which is about 2.5 folds of the incidence risk of 24.07%(13/54) in the other patients (P = 0.022). This result implied that the co-expression of NTS and IL-8 might be a valuable predictive factor for HCC prognosis.

**Table 2:** The correlation between the co-expression of NTS and IL-8n in 64 cases of HCC tissues and multiple clinical-pathological features of the corresponding patients.

Clinic-pathological features		Case	Expression of NTS		*P* value	Co-expression of NTS and IL-8		*P* value
			−	+		−	+	
Gender	Female	6	6	0	0.241	6	0	0.268
	Male	58	47	11		48	10	
Age	<54.44 y	35	29	6	0.992	30	5	0.746
	≥54.44 y	29	24	5		24	5	
HBV infection	No	16	14	2	0.566	14	2	0.691
	Yes	48	39	9		40	8	
Alcohol history	No	33	28	5	0.656	28	5	0.914
	Yes	31	25	6		26	5	
Smoke history	No	26	22	4	0.752	23	3	0.456
	Yes	38	31	7		31	7	
Clinical stage of disease	I	8	6	2	0.805	6	2	0.681
	II	45	38	7		39	6	
	III	11	9	2		9	2	
Clinical outcome	Alive	45	40	5	**0.047**	41	4	**0.022**
	Dead	19	13	6		13	6	

Note:

−: low expression of NTS or negative of co-expression of NTS and IL-8 in cancer tissues;

+: high expression of NTS or positive of co-expression of NTS and IL-8 in cancer tissues.

**Figure 8:**
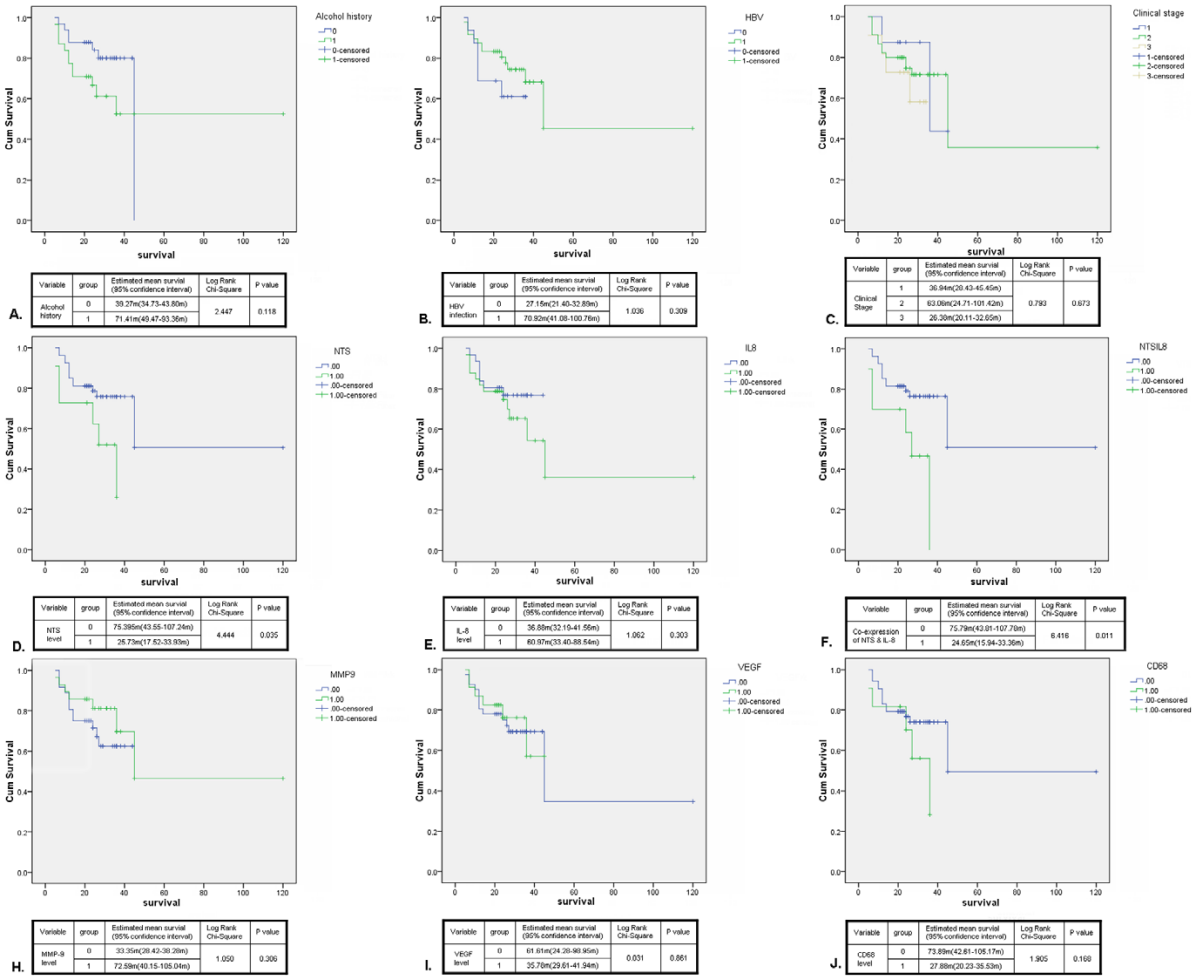
Synchronous increase of NTS and IL-8 in HCC correlated with worse prognosis and shorten survival of patients after surgery. The univariate survival analysis between different clinical and pathological parameters of 64 cases of HCC patients was performed using the Kaplan-Meier method and the log-rank test. A-C). None significant difference of OS was observed between patients with and without history of alcohol consumption(A) or HBV infection(B), and patients at different clinical stages(C). D-J). The relationship between the OS and the expression levels of NTS(D), IL-8(E), MMP-9(H), VEGF(I) and CD68(J) was studied. The univariate survival analysis showed that the expression level of NTS exclusively adversely affected the OS of HCC patients(D), in which the patients bearing NTS^+^ tumors suffered from shorter OS than those bearing NTS^−^ tumors (P = 0.035). The same result was obtained in the patients bearing NTS^+^IL-8^+^ tumors (F) whose OS are 3 folds shorter than the others(P = 0.011). Note: 0: Negative/low expression of certain markers in cancer tissues; 1: Positive expression of certain markers in cancer tissues.

Therefore, the univariate survival analysis between different clinical and pathological parameters and OS of HCC patients was performed using the Kaplan-Meier method and the log-rank test. As shown in Fig. 8, no significant difference of OS was observed between patients with different gender, age, status of HBV infection, history of alcohol consumption or clinical stages (Fig. 8A–C). Next, we evaluated the relationship between the OS and the expression levels of NTS, IL-8, MMP-9, VEGF and CD68 (Fig. 8D–J). The univariate survival analysis showed that the expression level of NTS exclusively executed significant influence on the OS of HCC patients, in which the NTS^+^ HCC patients suffered from shorter OS than the NTS^−^ HCC patients (P = 0.035, Fig. 8D). The same result was observed in the patients bearing NTS^+^IL-8^+^ tumors whose OS are 3 folds shorter than the others (P = 0.011, Fig. 8F). In order to distinguish the independent predictive factors of OS, the multivariate COX regression analysis was performed. It’s demonstrated that the co-expression of NTS and IL-8 in cancer tissues is an independent prognostic factor to predict the OS of HCC patients. Compared to the HCC patients without co-expression of NTS and IL-8, the OS of NTS^+^IL-8^+^ HCC patients significantly decreased (24.65±4.45 m vs. 75.79±16.32 m, P = 0.013), and these patients are under a higher hazard of death at an expected hazard ratio (HR) of 3.457.

## Discussion and Conclusions

Chronic inflammation has been recognized as a pivotal pathological factor in the development of hepatitis-related HCC. But the role of ongoing inflammation in disease progression has not yet been elucidated. Some studies have demonstrated that the metastatic potential of HCC is influenced by the local inflammatory microenvironment, in which the pro-inflammatory peritumoral cytokine milieu and increased infiltration of neutrophils in the stroma may play significant roles in promoting the metastatic spread of HCC [27], [28]. However, our understanding of the key molecular mechanisms and predominant signaling pathways that regulate this pathological process is limited. Utilization of high-density nucleic acid microarrays is one of the most effective approaches to identifying these key molecular events. In this study, we used the GeneChip Human Genome U133 plus 2.0 arrays and identified a specific subgroup of HCC displaying significantly enhanced inflammatory properties in which up-regulated expression of NTS was revealed as the characteristic molecular phenotype. In order to corroborate this result, three public expression profiling data sets from GEO were utilized and integrated into our in-house data to repeat the comprehensive bioinformatics analysis. Accordingly, a specific subgroup of HCC displaying significantly activated inflammation-related pathways and up-regulated NTS expression was observed, accompanied by activation of two well-known invasion-related pathways: HIF-1α signaling and Wnt/β-catenin signaling. Therefore, our results implied that NTS might represent a key protein biomarker to induce and maintain an inflammatory tumor microenvironment and promote tumor invasion and metastasis in a certain subgroup of HCC.

NTS is a 13 amino acid neuropeptide first isolated from the bovine hypothalamus by Carraway et al. in 1973 [29]. NTS is widely expressed in the brain and gastrointestinal tract of humans and animals. As a neuroendocrine peptide, NTS has significant interaction with the dopaminergic system and plays an important role in lowering blood pressure and regulating gastrointestinal functions [23]. In recent years, some studies have demonstrated that NTS can provide a growth signal to multiple types of epithelial-derived malignancies including colon cancer, pancreatic cancer, breast cancer, lung cancer, prostate cancer and head and neck cancers [26], [30]–[32]. The engagement of the high affinity receptor NTR1 with NTS lead to the activation of the phospho-inositide and adenylyl cyclase pathways which directly stimulate the proliferation, migration and invasion of various cancers cells and facilitates cancer growth [33]. In addition, multiple downstream signaling pathways are activated after interaction of NTS and NTR1, including the mitogen-activated protein kinases (MAPKs), extracellular signal-regulated kinases (ERK), c-Jun N-terminal kinases (JNK), RhoGTPase and NF-κB, resulting in the expression of a number of inflammation-related genes, such as IL-8 in certain solid tumors like colorectal cancer, pancreatic cancer, prostate cancer and head and neck cancer etc. [34]. Carraway *et al.* [35] speculated that NTS induced IL-8 secretion after activation of the NTS/IL-8 pathway resulted in the formation of an inflammatory microenvironment, which was confirmed by subsequent studies in colon cancer where an activated NTS signaling pathway definitely aggravated local inflammation and promoted EMT of cancer cells via TGF-β signaling [36].

IL-8 (CXCL8), a pro-inflammatory cytokine secreted by cancer cells and macrophages and a chemotactic factor for leukocytes, is reported to promote tumor invasion, progression and angiogenesis in multiple solid tumors, including colorectal cancer, prostate cancer, breast cancer, non-small cell lung cancer, pancreatic cancer, ovarian cancer and bladder cancer [24]. The biological effects of IL-8 are mediated through the binding of IL-8 to CXCR1 and CXCR2, two G protein coupled receptors (GPCRs) expressed on cancer cells, endothelial cells, neutrophils and TAMs. Intratumoral IL-8 expression is proposed to be a key regulator enhancing angiogenesis [37] and infiltration of neutrophils and TAMs *in situ* [38]. It also increases proliferation, migration and invasion capacity of tumor cells [39]–[41] and maintains self-renewal and survival of cancer stem cells [42] which favors the generation of a metastasis-prone microenvironment. Thus, inhibiting the expression of IL-8 in tumor cells by blocking NTS stimulation has been accepted to represent an effective interventional strategy of anti-cancer therapy in colorectal cancer [43]. Therefore, we proposed that over-expression of NTS in HCC induces the development of inflammation and promotes tumor invasion by inducing the secretion of IL-8 via the NTS/IL-8 pathway.

NTS is frequently expressed in the gastrointestinal tract but rarely detected in HCC. Normally, NTS is transiently expressed in fetal liver and disappears from normal mature liver and most of primary HCC tissues [44]. In our study, IHC results demonstrated that the NTS protein is predominantly expressed on the membrane and in the cytoplasm of cancer cells rather than stromal cells, implying that apparent ectopic expression of NTS exists in some HCC samples. Accordingly, Tang et al. reported recently that up-regulated NTS expression was detected in some HCC cell lines [45] which induced secretion of IL-8 and promoted CD133^+^ liver tumor-initiating cells (TICs) exhibiting a greater ability to self-renew. Consistently, both our microarray data and IHC results demonstrated that the expression of NTS both at RNA level and protein level is positively correlated with IL-8, suggesting that the NTS/IL-8 pathway is abnormally activated in some HCC samples.

In order to determine if the activation of NTS/IL-8 pathway in HCC induces the inflammatory microenvironment as previously observed in colorectal cancer, the expression of predominant downstream inflammation-related biomarkers, such as VEGF and MMP9, and infiltration of inflammatory leukocytes (TAMs) were detected in high NTS-expressing HCC samples. We demonstrated that the expression levels of VEGF and MMP9 were significantly correlated with co-expression of NTS and IL-8 in HCC. Similarly, increased infiltration of CD68^+^ TAMs was observed in NTS^+^IL-8^+^ samples, which implied that activation of NTS/IL-8 is prone to promote the development of an inflammatory microenvironment in HCC.

Furthermore, since intratumoral IL-8 expression is reported to increase the invasive potential of tumor cells, we compared the features of EMT between high and low NTS-expressing HCC samples. EMT is a biological process that allows a polarized epithelial cell to undergo multiple biochemical changes that enable it to assume a mesenchymal phenotype, which has been confirmed as an important molecular basis for the recurrence and metastasis of epithelial-derived cancers [46]–[48]. As the characteristic features of EMT, lost expression of E-Cadherin on the membrane and increased accumulation of β-catenin and vimentin in the cytoplasm were exclusively observed in NTS^+^IL-8^+^ HCC samples which implied that dysfunctional activation of NTS/IL-8 pathway rather than ectopic expression of NTS alone is essential for the development of an invasive phenotype in HCC cells. Further survival analysis demonstrated that the co-expression of NTS and IL-8 in HCC significantly correlated with worse prognosis and shorter survival of patients after surgery, suggesting that dysfunctional activation of the NTS/IL-8 pathway significantly promotes progression of disease and adversely affects prognosis of HCC patients after surgery.

Thus, our results point out that the dysfunctional activation of a specific NTS/IL-8 pathway in a certain subgroup of HCC could be responsible for worse outcomes by promoting the development of a local inflammatory microenvironment and increasing the invasive potential of tumor cells which might be proposed as a promising therapeutic target and a predictive biomarker for HCC diagnosis and treatment.
